# Preparation and Evaluation of Composite Hydrogel for Reducing the Leakage Rate of Lost Circulation

**DOI:** 10.3390/polym15214218

**Published:** 2023-10-25

**Authors:** Qisheng Jiang, Peng Xu, Jie Xu, Manfu Hou, Qinglin Liu, Baimei Dai

**Affiliations:** 1 Cooperative Innovation Center of Unconventional Oil and Gas, Yangtze University, Wuhan 430100, China; 2021720454@yangtzeu.edu.cn; 2 School of Petroleum Engineering, National Engineering Research Center for Oil & Gas Drilling and Completion Technology, Yangtze University, Wuhan 430100, China; 2021720455@yangtzeu.edu.cn (Q.L.); 2021720475@yangtzeu.edu.cn (B.D.); 3 Hubei Key Laboratory of Oil and Gas Drilling and Production Engineering, Yangtze University, Wuhan 430100, China; 4 Jingzhou Jiahua Technology Co., Ltd., Jingzhou 434023, China; 5 Haikou Marine Geological Survey Center, China Geological Survey, Haikou 571127, China; 6 Bohai Drilling International Engineering Company, Tianjin 300450, China; houmanfu@cnpc.com.cn

**Keywords:** lost circulation, fracture, PVA, sodium silicate

## Abstract

Fractured reservoirs are widely distributed and rich in hydrocarbon resources. When encountering fractured reservoirs during the drilling process, it is often accompanied by formation losses characterized by high leak-off rates, causing severe damage to the reservoir and hindering the detection of oil and gas layers, which is not conducive to the accurate and efficient development of the reservoirs. Conventional plugging materials have poor retention effects in the fractures, resulting in the low stability of the sealing layer. The treatment of malignant lost circulation in fractured formations is an urgent problem to be solved in drilling engineering. This article focuses on improving the success rate of formation plugging through the combined use of multiple plugging materials and develops a composite hydrogel that can effectively reduce leakage rates. This hydrogel is mainly composed of materials such as polyvinyl alcohol, borax, and sodium silicate. It has good temperature resistance, maintains good gel strength at 60 °C, and can maintain long-term performance stability under simulated geological water conditions with salinity of 12,500 mg/L. For immersion corrosion by water or gasoline, the amount of corrosion is small and its fundamental performance remains largely unchanged. Through indoor simulation of a leak formation scenario, the hydrogel demonstrates commendable sealing pressure-bearing capacity. In terms of delaying fluid leakage, mixing the hydrogel with cement slurry at a ratio of 1:1 can delay the leakage rate of the cement slurry by a factor of 5.29.

## 1. Introduction

With the focus of oil and gas reservoir development shifting towards deep, unconventional, and other complex resources, the issue of lost circulations has become one of the main factors restricting the precise and efficient development of reservoirs. In order to effectively control and address the problem of formation losses, practitioners in the oil and gas industry have developed various types of plugging materials, such as bridge plugging materials [1,2,3], gel plugging materials [4,5,6], and consolidated plugging materials [7,8]. However, when it comes to leakage in fractured reservoirs, conventional plugging materials are difficult to strand in the fracture, resulting in the low stability of the sealing layer. Therefore, the development of new plugging materials or sealing methods has become the key to scientifically and effectively addressing the issue of leakage in fractured formations [9].

To date, many scholars have achieved excellent research results in the study of plugging materials for reservoir leakage. Wu Xuepeng [10] developed a flexible expanding material that is resistant to high temperatures and can expand at 150 °C conditions, using materials such as flake graphite, ice acetic acid, and a high-chlorine-acid CL intercalating agent as the main raw materials. This material can effectively reduce the filtration loss of drilling fluid at high-temperature and high-pressure conditions, and it has high plugging strength after self-expansion. However, this material is at the nanometer level and is not a good choice for large cracked leakage formations. Chen Kang, Zhang Xiao, et al. [11] used materials such as acrylic resin and nitrile rubber to prepare a plugging material with particle surfaces that are sticky. The particles bond together at high temperatures, forming a sealing layer with certain pressure resistance in the cracks. However, the outer layer of this material is oil-soluble, and its ability to bond and seal will be compromised when it comes into contact with hydrocarbon fluids. Ahmad A. Adewunmi et al. [12] used polyacrylamide as the main raw material and polyethyleneimine as the crosslinking agent and introduced fly ash to improve the thermal stability and strength of the gel. However, its resistance to dilution is one of the key factors for its retention and completion of plugging in the formation.

Considering the cost, convenience, and applicability on-site, bridge plug plugging [13] is often chosen. By using particle matching [14,15] for different leakage channels, bridge plug materials are densely packed in the cracks to improve the pressure-bearing capacity of the leaking formations. However, with the advancement of oil and gas production, the proportion of fractured reservoirs in oil and gas resources is continuously increasing [16]. The deformation patterns of fractured formations are complex [17], and bridge materials have poor compatibility with the size of the leakage channels, resulting in ineffective plugging and the easy recurrence of leakages [18]. Therefore, we have adopted a research approach of using multiple plugging materials in combination to improve the success rate of plugging [19,20]. We have developed a composite hydrogel that can delay the rate of reservoir leakage. It provides a suitable plugging environment for other plugging materials used in conjunction with it, thereby increasing the success rate of formation plugging.

The reversible gel formed by PVA and borax has attracted widespread attention due to its excellent physicochemical properties and industrial value. Hsiu-Li Lin et al. [21] suggested, through light scattering experiments, that the diol complexation process between polyvinyl alcohol (PVA) and borax is a thermally reversible crosslinking process. Hasna Faten Mahjoub et al. [22], comparing the reactions of PVA with different mass fractions of borax, found that the concentration of borax required for gelation is different. By using an alkali treatment method, they confirmed that the addition of sodium hydroxide can convert the attached borate on the PVA chain into borate ions, resulting in diol complexation. The basic gel composition of the composite hydrogel consists of PVA, sodium silicate, and borax. The hydrolysis of sodium silicate provides an alkaline environment that promotes diol complexation between PVA and borax. Huang Peilin [23], through infrared spectroscopy analysis, found that an increase in the amount of sodium silicate promotes the chemical reaction between PVA and sodium silicate. In this study, by comparing the effect of sodium hydroxide and sodium silicate on gel strength, it is observed that the reaction between PVA and sodium silicate improves the strength and dilution resistance of the gel to some extent. Additionally, we used cellulose fibers and dibutyl phthalate to enhance the overall performance of the composite hydrogel. This article compares the performance of the gel (referred to as PMG hereafter) with commonly used gels in the field in terms of temperature resistance, erosion resistance, and salt resistance. The experimental results demonstrate that PMG exhibits excellent salt and dilution resistance. In the simulated formation leakage experiment, PMG performs well in all tested aspects. PMG can serve as an auxiliary plugging material to slow down the formation leakage rate and provide favorable plugging conditions for other plugging materials. This study can provide a reference for plugging methods in complex leaky formations with water.

## 2. Experimental Section

### 2.1. Material Selection

Experimental materials: polyvinyl alcohol (PVA) with a degree of polymerization of 1700 and an alcoholysis rate of 88%, purchased from Shanghai Chenqi Huagong Technology Co., Ltd., Shanghai China; sodium tetraborate decahydrate (borax), analytical grade, purchased from China National Pharmaceutical Group Chemical Reagent Co., Ltd., Shanghai China; dibutyl phthalate (DBP), analytical grade, China National Pharmaceutical Group Chemical Reagent Co., Ltd., Shanghai China; DuPont fiber, length of 2.0 cm, purchased from Shandong Shuntong Engineering Materials Co., Ltd., Shandong China; distilled water, prepared in the laboratory.

Experimental instruments: rotary agitator (Jincheng Guosheng Experimental Instrument Factory, Jiangsu, China); DV-II+ pro viscometer (Ametek LTD, California, USA); thermostatic water bath (Tianjin Saidelis Experimental Analysis Instrument Factory, Tianjin, China); gel strength tester (Lin’an Fengyuan Electronics Co., Ltd., Zhejiang, China); pressure reducing valve (Qingdao Huaqing Automation Instrument Co., Ltd., Shandong, China); pressure gauge (Xi’an First Automation Instrument Factory, Shaanxi, China); piston container (Nantong Yichuang Experimental Instrument Co., Ltd., Jiangsu, China); core gripper (Nantong Yichuang Experimental Instrument Co., Ltd., Jiangsu, China); confining pressure pump (Nantong Yichuang Experimental Instrument Co., Ltd., Jiangsu, China).

### 2.2. Preparation Process

The preparation of a composite hydrogel requires the prior preparation of high-concentration aqueous solutions of specific chemicals such as PVA, sodium silicate, and borax. The concentration of the prepared solutions depends on the desired composition of the final composite hydrogel. Here, we provide an example of the preparation of a composite hydrogel with 200 g containing 3.0% PVA, 1.0% sodium silicate, 0.6% borax, 0.3% dibutyl ester, and 0.5% Dura fiber. The process of preparing the composite hydrogel is as follows.

(1) In a three-necked flask, add 285 g distilled water. Heat a water bath to 80–90 °C, start the stirrer, and slowly add the PVA powder in portions to prevent insufficient dissolution due to clumping. Keep heating and stirring after all the powder has dissolved. Remove it after 1 h and let it cool for later use.
(2) In a beaker containing 197 g distilled water, add 3 g of borax, thoroughly stir, and set aside.
(3) Take 120 g of the solution prepared in step (1), add 2 g sodium silicate, stir well, and then add 0.6 g of dibutyl ester and 1.0 g of Dura fiber. Stir well and then add 80 g of the solution prepared in step (2). Continue stirring slowly until a uniform gel is formed.

### 2.3. Method Selection

(1) Gel strength testing. Gel strength was evaluated through visual observation using Sydnsk’s Gel Strength Codes (GSC) [24]. The details of the visual observation codes are provided in Table 1. The test was conducted using a cylindrical glass bottle with a volume of 150 mL and a length of 20 cm. First, 30 mL of gel was taken and allowed to stand in the bottle for five minutes. The flow state of the gel was observed by inverting the bottle, and the strength was judged. The experiment was repeated three times to confirm the observations.
(2) Thermal resistance testing. The gel samples were heated in a water bath to the test temperature and then measured using a gel strength tester. To test the gel strength using a gel strength tester, the gel needs to be placed in the testing container with a thickness of 5 cm. The gel strength is measured by recording the pressure displayed on the instrument when the metal rod of the gel strength tester is pressed into the gel to a depth of 4 cm. The experiment is repeated three times, and if there is a significant fluctuation in the results, additional tests are conducted and any results with significant deviations are excluded. Finally, the average value is calculated.
(3) Salt resistance testing. Different salinity saltwater formulations were prepared according to the mass ratio of NaCl:CaCl_2_:MgCl_2_·6H_2_O = 7:0.6:0.4 to evaluate the gel’s salt resistance performance. Take 200 mL of gel, crush it into small pieces, and mix it with saltwater in a 1:1 ratio. Stir the mixture at a speed of 120 r/min for 20 min at room temperature. Then, separate the gel from the saltwater and measure its remaining volume and strength. Repeat the process twice and calculate the average of the results.
(4) Erosion resistance testing. Observe the volume changes of the gel when in contact with different fluids to assess the compatibility between the gel and formation fluids. Take 200 mL of gel, crush it into pieces, and then place it in a beaker. Add sufficient water or oil to the beaker to ensure complete immersion of the gel. Record the remaining mass of the gel at different time intervals.

Plugging performance testing. The gel blocking performance was characterized using an indoor experimental apparatus consisting of a non-permeability drilling fluid filtration tester, steel-fractured core samples, and intermediate containers to simulate a leakage environment. The gel’s blocking performance was evaluated based on its delay effect and pressure-bearing capacity.

## 3. Results and Discussion

### 3.1. Plugging Mechanism of PMG

Fractured formations with leakage often exhibit high leakage rates. Traditional plugging materials have difficulty staying in the leakage channel. The correct approach to addressing leakage in fractured formations is to reduce the leakage rate using a certain method and then seal the leaking formation with traditional plugging materials. Gel-like materials are less restricted by the crack morphology and can be used to temporarily block or delay the extent of formation leakage, providing better conditions for subsequent plugging materials. The PMG developed in this study can quickly form a highly viscoelastic gel, which facilitates pumping. The PMG can be easily mechanically crushed, and the gel fragments are squeezed into the cracks, gradually filling the entire crack. The crushed PMG accumulates in the cracks and re-fuses into a continuous whole, forming a gel plug with a certain compressive strength. This gel plug isolates the drilling fluid from the formation fluid, temporarily blocking or segregating the formation fluids, and provides favorable conditions for other types of plug materials.

**Figure 1:**
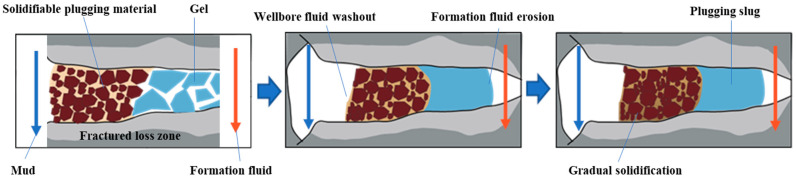
PMG combined with consolidated materials for plugging.

Figure 1 illustrates the schematic process of plugging using PMG in combination with consolidated plugging materials. The drilling fluid carries PMG into the leaking formation, and the PMG fragments accumulate and remain in the leakage channel. Once the gel plug is formed, the consolidated material will not lose its curing condition due to dilution by the formation fluid. It slowly cures in the channel and eventually forms the sealing layer.

**Figure 2:**
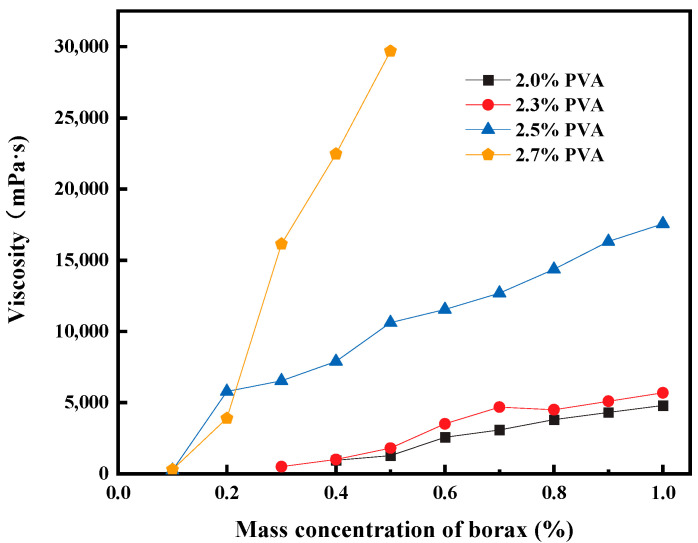
Crosslinking results of different concentrations of PVA and borax.

Mixing PVA with borax can result in a highly viscous and flowable gel (hereafter referred to as PVA gel). Figure 2 shows the variation in gel viscosity as PVA aqueous solutions with different concentrations are crosslinked with borax. When the viscosity of the PVA gel exceeds 15,000 mPa·s, the gel strength gradually transitions into a flowable gel. As shown in Figure 2, low-concentration PVA solutions do not show a significant increase in viscosity with an increase in the amount of borax. Conversely, high-concentration PVA aqueous solutions can generate high-viscosity gels with only a small amount of borax.

The reaction between polyvinyl alcohol and borax is considered to involve two reactions [21,25], i.e., monodiol complexation (Reaction (1)) and a crosslink reaction (Reaction (2)), as shown by the following equations:


(1)

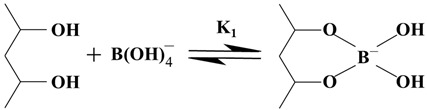




(2)

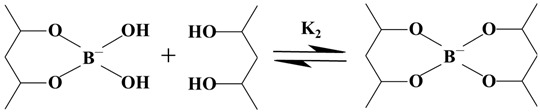



The poor anti-dilution performance and high fluidity of PVA gel make it ineffective in sealing complex water-bearing formations. The introduction of sodium silicate into the PVA gel can improve its mechanical strength and, to some extent, enhance its erosion resistance to formation fluids. This is due to the alkaline environment formed by the hydrolysis of sodium silicate, which promotes the diol complexation of polyvinyl alcohol (PVA) and borax, thereby increasing the gel strength. By comparing the effects of sodium hydroxide and sodium silicate on the strength of PVA gel and analyzing the infrared spectra, we have determined that sodium silicate not only promotes the diol complexation between PVA and borax, but also reacts with PVA. The reaction between sodium silicate and PVA has a positive effect on the strength of the PVA gel. Table 2 describes the strength of the PVA gel under different experimental conditions.

**Figure 3:**
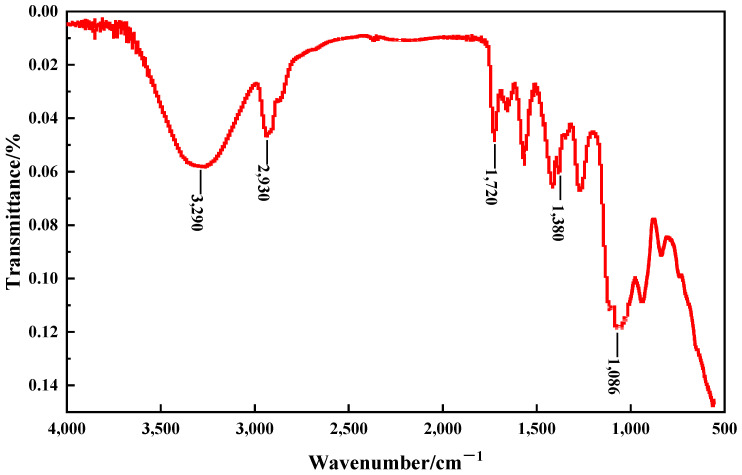
FT-IR spectra of: 3.0% PVA + 1.0% sodium silicate + 0.6% borax.

To further confirm the components of PMG, an infrared spectroscopic analysis was conducted on a hydrogel made from the most fundamental gel constituents of PMG, namely 3.0% PVA, 1.0% sodium silicate, and 0.6% borax. In Figure 3, the peak near 3200 cm^−1^ corresponds to the stretching vibration of -OH, while the peak at 1720 cm^−1^ represents the stretching vibration of C=O. The peak at 1240 cm^−1^ indicates the asymmetric stretching vibration of C-O-C, and the peaks at 1380 cm^−1^ and 2930 cm^−1^ correspond to the asymmetric stretching vibrations of B-O-C and C-H in the CH2 groups, respectively. The absorption peak near 1086 cm^−1^ is attributed to the Si-O-C bonds. These findings confirm that both sodium silicate and borax undergo chemical reactions with PVA.

### 3.2. Preparation of PMG

The strength, stability, and pumpability of the plugging gel are closely linked to construction safety and the formation sealing effectiveness. The indoor preparation of PMG is conducted using high-concentration agents. The gel possesses strong viscoelasticity and can be easily mechanically fractured, gradually healing into a continuous colloid under static or temperature effects, ensuring the pumping performance and sealing capability of the gel. Different dosages of reagents will have varying effects on the gel’s performance. This section primarily focuses on studying the formulation and determining the dosage of PMG.

#### 3.2.1. Effect of Borax on PMG

The main function of borax is to provide borate ions, which, when combined with polyvinyl alcohol, form a three-dimensional network structure and result in a stronger gel network in an alkaline environment. The dosage of borax should be controlled between 0.3% and 0.8%. An adequate amount of borax is necessary to fully crosslink the polyvinyl alcohol and form a hydrogel with certain strength. Excessive amounts of borax will lead to a rapid reaction with partially crosslinked materials, causing the localized formation of highly viscous and elastic colloids, making it difficult to form a uniform hydrogel.

#### 3.2.2. Effect of Polyvinyl Alcohol on Strength of PMG

The experimental results obtained from crosslinking experiments using only PVA and borax show that when the PVA content is less than 2%, as the amount of borax increases, the gel viscosity increases but the viscosity remains relatively low. The gel strength is measured using the visual observation method, and the gel strength formed within this dosage range falls between A and B. Its viscosity variation can be seen in Figure 2.

**Figure 4:**
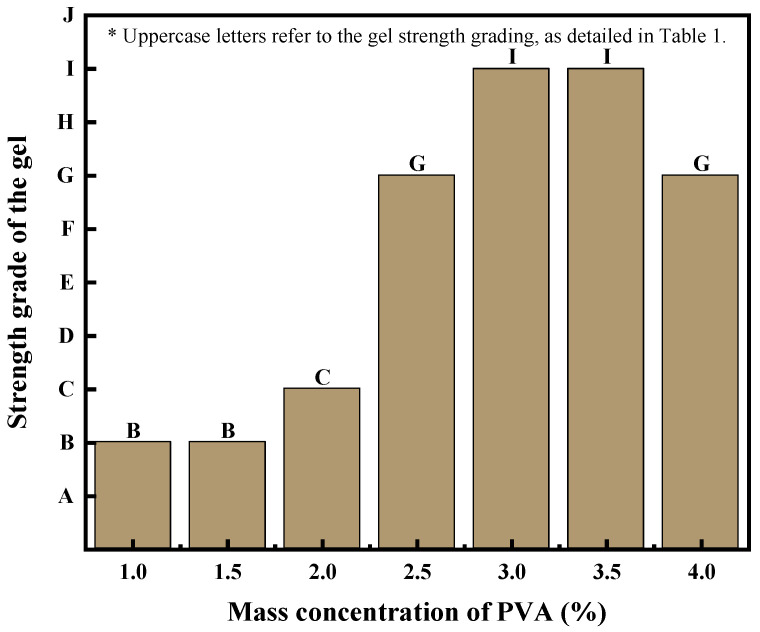
Effects of sodium silicate on PVA gel.

The erosion resistance and strength of the PVA gel can be enhanced with the addition of sodium silicate. Figure 4 illustrates the experimental results of PVA with different mass concentrations combined with 1% sodium silicate and 0.6% borax. At a mass concentration of 4.0% PVA, the gel displayed local rapid crosslinking, resulting in uneven gel strength.

#### 3.2.3. Effect of Sodium Silicate on Strength of PMG

The reaction between sodium silicate and PVA generates Si-O-C bonds, which, in the macroscopic behavior of the gel, increases the strength of PMG while significantly reducing its fluidity. Additionally, the erosion resistance of PMG is also enhanced, effectively ensuring the stability of PMG’s performance during pumping and blocking processes. When the dosage of sodium silicate approaches 2%, PMG placed indoors for several hours exhibits varying degrees of color change, transforming from colorless and translucent to white, accompanied by different levels of dehydration. This renders it incapable of long-term storage.

**Figure 5:**
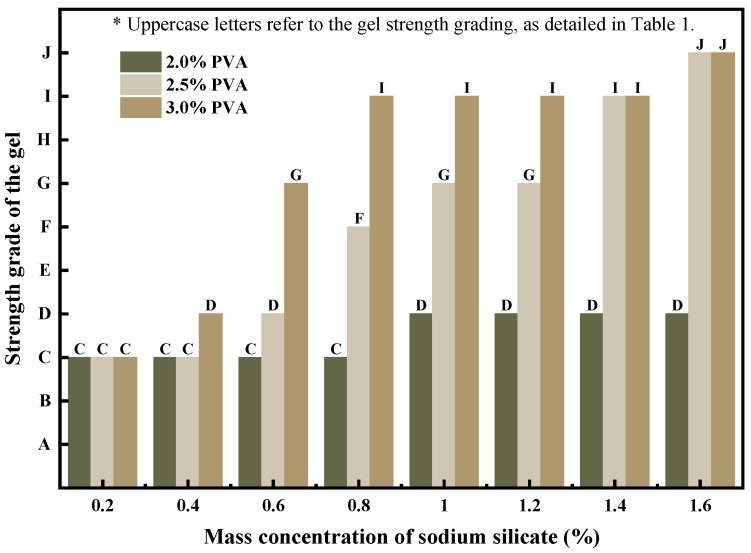
Effect of sodium silicate on gel strength.

In Figure 5, the strength evaluation of gels formed by different dosages of sodium silicate is depicted using the strength observation method.

#### 3.2.4. Effect of Dibutyl Ester on Strength of PMG

**Figure 6:**
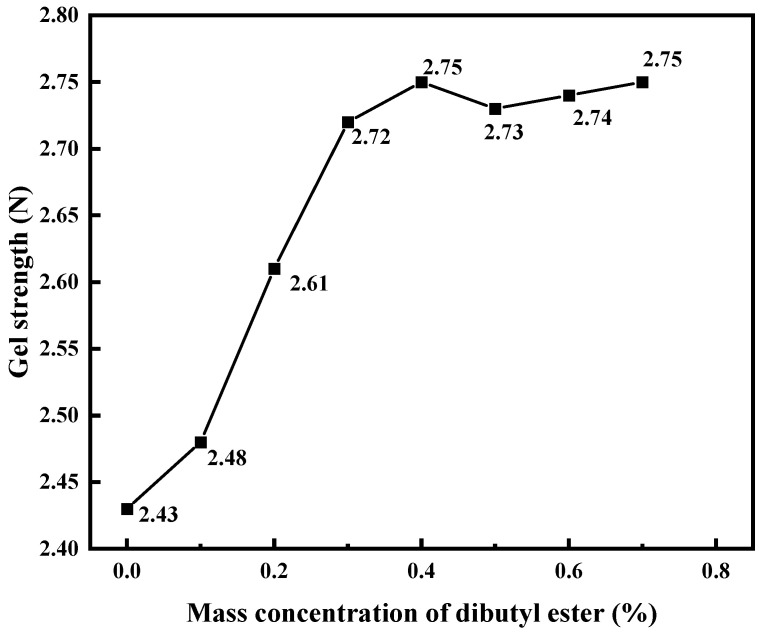
Effect of dibutyl ester on gel strength.

Phthalate plasticizers act between polymer chains, making them easier to slide and move, or by filling the gaps between polymer chains to enhance the flexibility of the polymer. We used dibutyl phthalate to improve the performance of PMG. Based on the experimental results shown in Figure 6, we ultimately selected a mass concentration dosage of 0.3%.

#### 3.2.5. Effect of Dura Fiber on the Strength of PMG

**Figure 7:**
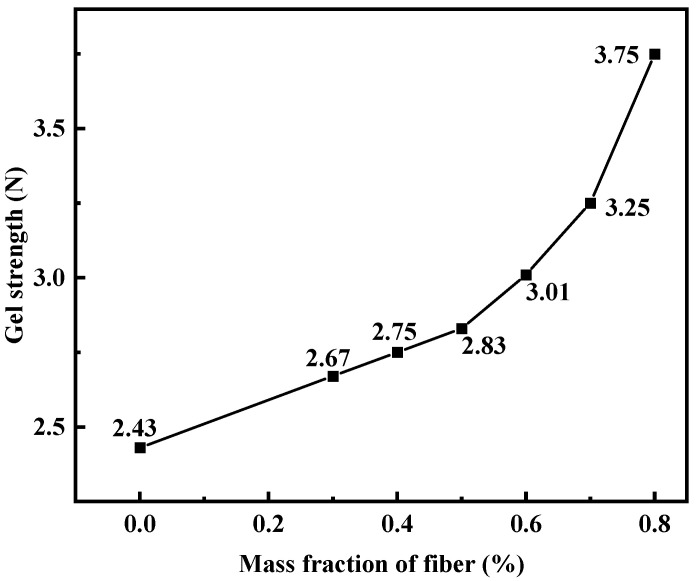
Effect of Dura fiber on gel strength.

When adding Dura fiber to the gel, it is necessary to consider whether its dosage can be sufficiently dispersed within the gel. Additionally, in order to facilitate its breakage into smaller pieces for easier pumping, the fiber dosage should not be too high. Dura fiber has a certain level of flexibility and can be effectively incorporated into the gel. However, when the dosage reaches 0.7%, fiber aggregation may occur, resulting in uneven gel strength. Figure 7 shows the results of the impact of the fiber dosage on gel strength. To optimize plugging, a fiber dosage of 0.5% was ultimately chosen.

Based on the analysis conducted, the optimal composition for the PMG can be determined as follows: 3.0% PVA, 1.0% sodium silicate, 0.6% borax, 0.3% dibutyl ester, and 0.5% Dura fiber.

### 3.3. Performance Evaluation of PMG

To evaluate the performance and efficacy of the composite hydrogels, the following six types of gels were prepared and tested under different conditions.

Gel I: 3.0% PVA + 1.0% sodium silicate + 0.6% borax.

Gel II: 3.0% PVA + 1.0% sodium silicate + 0.6% borax + 0.3% dibutyl ester.

Gel III: 3.0% PVA + 1.0% sodium silicate + 0.6% borax + 0.5% Dura fiber.

Gel IV: 3.0% PVA + 1.0% sodium silicate + 0.6% borax + 0.3% dibutyl ester + 0.5% Dura fiber.

Gel V and Gel VI are commonly used gels in oilfield operations, and the main component of these gels is a polymer material. Gel V and Gel VI are crosslinked using different crosslinking agents, respectively.

**Figure 8:**
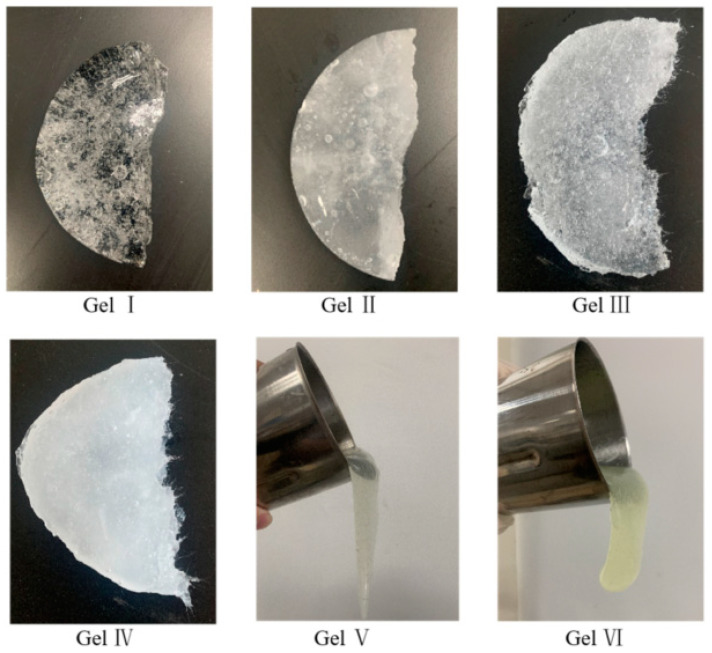
Appearance of different gels.

The appearance of the six tested gels is shown in Figure 8. Gel I has a relatively clear and bright surface, while gel II and gel IV, which have dibutyl phthalate added, exhibit a milky white color. Gel III and Gel IV, which have flexible fibers added, show needle-like structures at the fracture site. Gel V and Gel VI have high fluidity and are difficult to form into blocks. The picture shows their appearance when poured out of a cup, suspended in the air.

#### 3.3.1. Temperature Resistance of PMG

The above six different gels were prepared and placed in a thermostatic water bath for a duration of at least 1 h. After removal from the water bath, the gels were thoroughly mixed, and their temperatures and gel strengths were measured. A comparison was made to evaluate the influence of temperature on the strength of the different gels.

**Figure 9:**
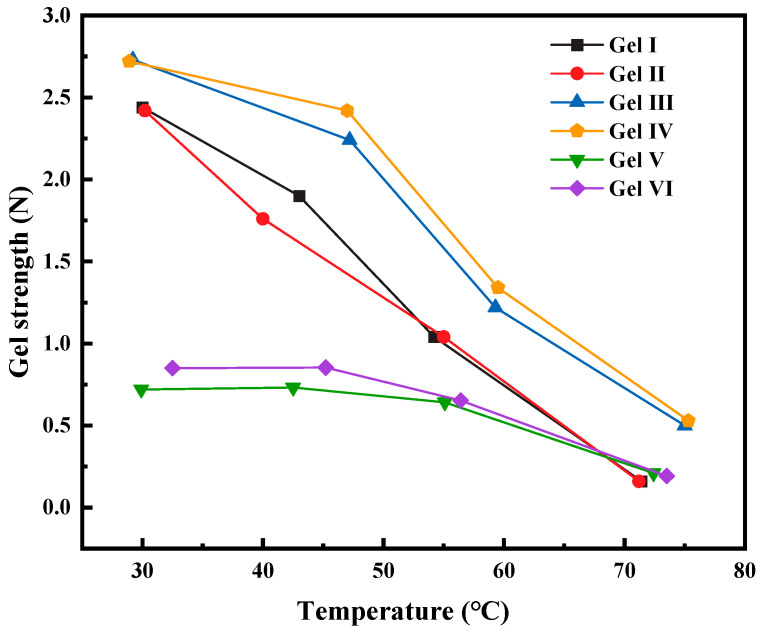
Effect of temperature on different gels.

According to the experimental results (see Figure 9), when comparing PMG with different compositions to the commonly used gel in the field, it is observed that the strength of the gels decreases as the temperature rises. Gel V and gel VI are less affected by temperature at lower temperatures, but their strengths are still lower than that of PMG. At higher temperatures, PMG containing fibers and dibutyl ester exhibits better thermal stability. Although it becomes unstable at high temperatures and its colloidal fluidity increases, it still maintains high viscosity. This can meet the plugging requirements for some leaking formations.

#### 3.3.2. Erosion Resistance of PMG

When the gel is broken and enters the formation, it comes into contact with formation water or crude oil, etc. The good anti-dilution ability of PMG is an important property that reduces the degree of formation leakage during plugging, which can ensure that PMG maintains high strength when transported to the leaking formation and in the leaking channel. This further enables the PMG to pile up, heal, and form a cohesive gel with a certain strength, separating the formation fluid and providing an excellent plugging environment for subsequent plugging materials to be used in conjunction.

**Figure 10:**
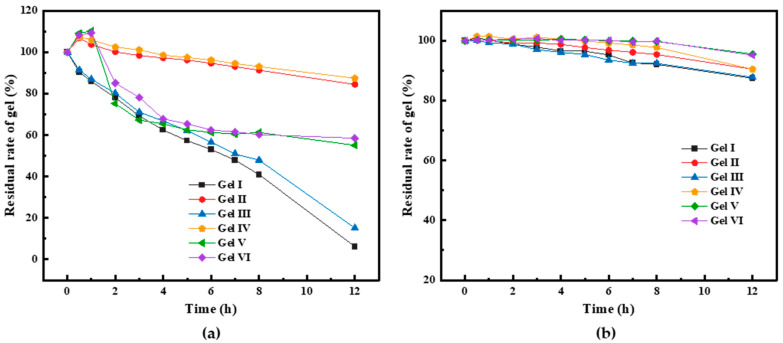
Erosion resistance of different gels: (**a**) shows the experimental results of the emulsification test using water as the soaking liquid; (**b**) presents the experimental results of the soaking test using gasoline as the soaking liquid.

From Figure 10a, gel V and gel VI have a certain degree of swelling ability. Within the first 5 h, their volume decreases by almost 40%. Gel V and gel VI absorb a lot of water, which results in high fluidity and low strength. The visual code indicates their strength as C, while the PMG codes for different components are E or above. In some cases, due to gel dissolution, the fiber content increases and the PMG strength becomes very high. Comparing PMG with different compositions, it can be observed that the gel with the addition of dibutyl phthalate exhibits much better anti-dilution performance compared to the gel without dibutyl phthalate. Additionally, when comparing gel I with gel III or gel II with gel IV, it can be noted that the gel with added fibers has a significantly lower loss rate compared to the gel without fibers. During the experimental process, due to prolonged immersion in the fluid, the surface of the gel becomes softer, more prone to dissolution, or susceptible to being washed away by the fluid. If fibers are added to the gel, when the fibers gradually become exposed on the gel surface, the contact area between the fluid and the gel decreases, which helps to slow down the reduction in gel volume. PMG can come into contact with aliphatic hydrocarbon components when it enters the formation. We submerged six types of gels in gasoline using identical experimental procedures. After 12 h of immersion in gasoline, the volume loss of all gels was less than 5%. Detailed results can be seen in Figure 10b.

When using formation water or highly mineralized water as the solvent to prepare the composite hydrogel, ions such as Na^+^, Ca^2+^, and Mg^2+^ combine with the free hydroxyl groups in polyvinyl alcohol molecules, reducing the crosslinking sites for borax and resulting in decreased gel strength. Among these ions, Ca^2+^ and Mg^2+^ react with the hydrolysis products of sodium silicate to generate precipitates, which can severely affect the strength and preservation of the gel. Therefore, when using highly mineralized water as the solvent, pre-treatment of the water is required.

For gel V and gel VI, there were no apparent changes within the first 20 min. However, after two hours of immersion in solutions with higher mineralization levels, the dehydration phenomenon became more evident. Table 3 provides a description of the salt resistance test conducted for gel IV.

**Figure 11:**
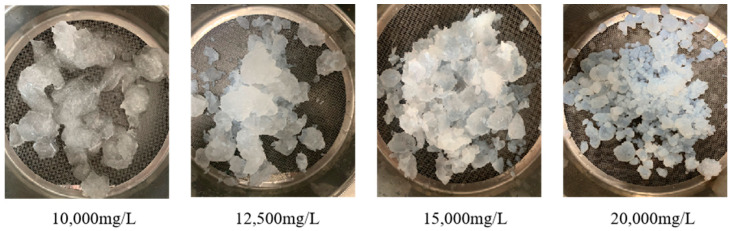
Apparent state of gel under different salinity.

Combining Table 3 and Figure 11, it can be observed that at low mineralization levels, the gel exhibits a reduction in dissolution, and, due to the increased relative content of fibers, the soaked gel shows a significant increase in strength. At high mineralization levels, the composite hydrogel reacts with metal cations, resulting in the whitening of the gel surface and shrinkage of the gel volume. Even after being partially removed from the saline solution, it continues to dehydrate, causing a severe reduction in gel volume. As a result, the gel loses its original flexibility and shows an inability to re-merge into a whole.

### 3.4. Evaluation of Plugging Performance

Currently, there are no corresponding testing standards for gel plugging materials. In this study, commonly used instruments in indoor experiments for bridging plugging materials, such as drilling fluid filtration testers and steel-cracked core samples, were utilized to simulate the leakage environment and assess the pressure sealing and plugging effectiveness of the gel.

#### 3.4.1. Evaluation of Leakage Rate Reduction Effect

**Figure 12:**
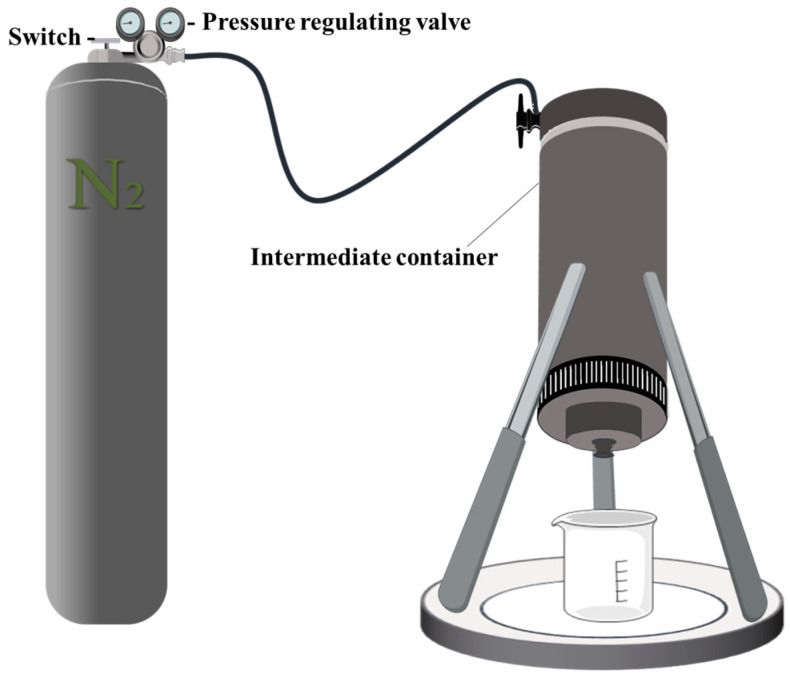
Reducing effect evaluation device.

The function of PMG is to reduce the leakage rate and provide a favorable plugging environment for other plugging materials, thereby improving the success rate of plugging. The experiment used an intermediate container without a piston, with a pipe cross-sectional area of 23 cm^2^ and an outlet radius of 2.2 mm. The delay effect of PMG was evaluated by comparing the time required for the pressurized fluid to be squeezed in the device under 1 MPa pressure. A simplified schematic diagram of the evaluation instrument is shown in Figure 12.

**Figure 13:**
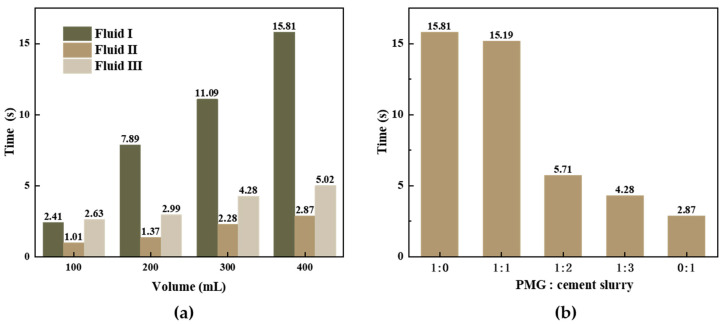
Evaluation of the leakage rate delay effect of PMG. Fluids I and III in (**a**) refer to PMG and cement slurry, respectively. Fluid II represents a mixture of PMG and cement slurry, where the x coordinate only indicates the volume change of the cement slurry for Fluid II. Each test of Fluid II contained an additional 100 mL of PMG. (**b**) shows mixes of PMG and cement slurry with different ratios, with a total volume of 400 mL.

The results in Figure 13a indicate a significant difference in fluidity between Fluid I and Fluid II compared to PMG. In each composition of Fluid III, 100 mL of PMG is quantitatively added. The experimental results show that PMG can delay its discharge rate in cement slurry. Figure 13b presents tests conducted on a mixture of 400 mL PMG and cement slurry with different ratios. The combination of PMG and cement slurry in a 1:1 ratio took only 5.29 times longer than the time taken for experiments conducted solely with cement slurry. When comparing experiments with ratios of 1:0 and 1:1, the timings are very similar. During the experiment, PMG is not completely discharged, but, rather, after the gel layer breaks, it forms a pathway through which gel fragments and cement slurry can pass. The final timing recorded for gas discharge marks the end of the measurement. In practical applications, consideration should be given to the compatibility of PMG with other fluids to achieve the optimal mixture and effective sealing.

#### 3.4.2. Simulated Sealing in Fractured Formation

**Figure 14:**
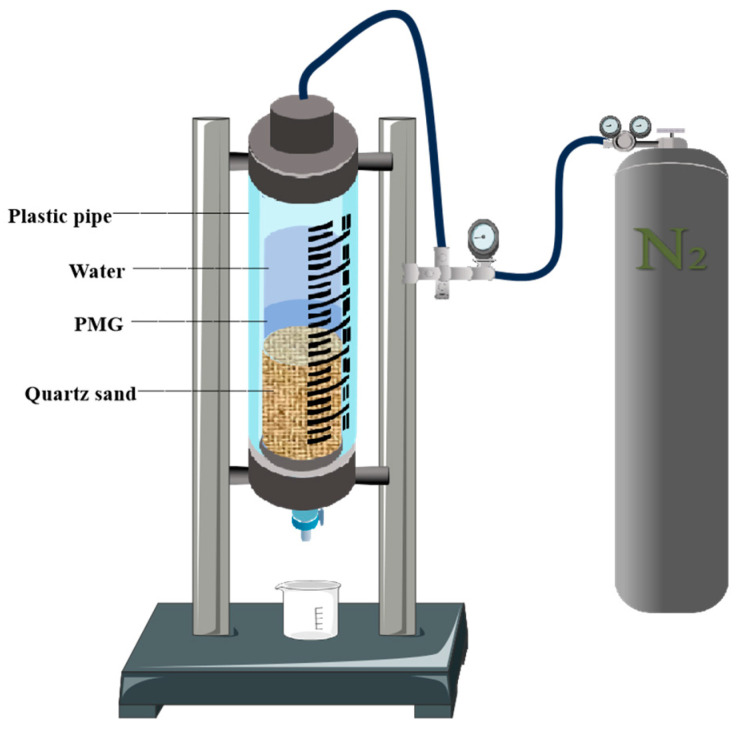
Drilling fluid filtration testers.

The effective filtration area of the drilling fluid filtration testers used in the experiment was 18 cm^2^. First, 350 cm^3^ of 40–70-mesh quartz sand was poured into it to simulate fractured formations. Different gel samples of 100 mL were taken and poured into the pipeline each time, followed by 200 mL of water. Then, the nitrogen valve was opened, and different pressure values were applied. If the pressure could be maintained for 30 min without any leakage, the experimental pressure was increased for retesting. The schematic diagram of the experimental apparatus used is shown in Figure 14.

**Figure 15:**
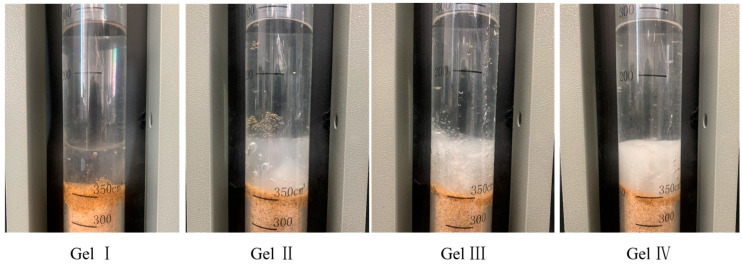
Simulation results of fractured formation plugging at 1.2 MPa.

The results are shown in Figure 15: all four gels were able to withstand pressure for 30 min under the maximum working pressure of 1.2 MPa in the instrument. As the test pressure increased during the experiment, both the sand layer and the gel were compressed, causing the water interface in the pipeline to move downward. However, all four gels were able to form effective sealing without any water permeating and flowing out of the sand layer. The difference lies in the fact that gel I and gel II exhibited more noticeable penetration into the quartz sand under the influence of pressure.

#### 3.4.3. Evaluation of Pressure Resistance Performance

**Figure 16:**
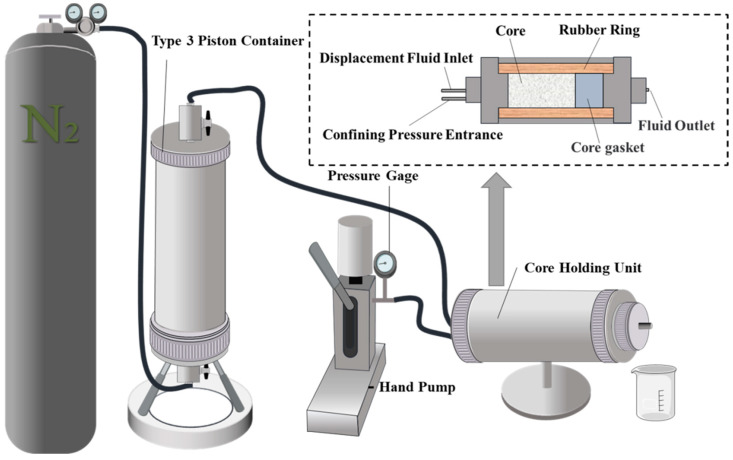
Pressure bearing simulation device for formation fracture plugging.

Using a steel-fractured core sample to simulate a crack leak channel, the core sample was 6 cm long with a diameter of 2.5 cm, and it simulated a wedge-shaped crack morphology. The sealing capacity experiment was conducted using a leak-plugging evaluation device, which mainly consisted of a nitrogen cylinder, pressure regulator, intermediate container, core holder, and confining pressure pump. The configuration of the device during the experiment is shown in Figure 16. During the experiment, the confining pressure was initially adjusted using a hand pump to prevent fluid from passing through the side of the core sample. Then, the pressure of the pressurization device was increased, and the occurrence of gel flow at the outlet end of the core sample was observed. If the water flow could still be blocked after 30 min, the pressure difference was further increased until the sealing could not be maintained.

**Figure 17:**
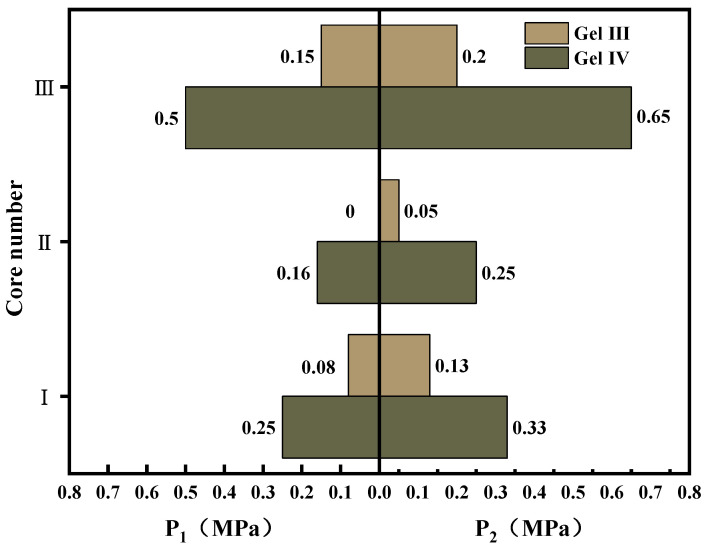
Test results of pressure bearing properties of gel III and gel IV. P_1_ refers to the experimental pressure under which the amount of gel extruded is less than 0.1 g. P_2_ refers to the experimental pressure under which the gel is squeezed out, but no other liquid flows out.

The experimental core fracture parameters (see Table 4) and experimental results (see Figure 17) are as follows.

The experimental results (see Figure 17) indicate the following: gel I and gel II have high gel mobility and do not possess the ability to withstand pressure in larger cracks. The results of the pressure performance tests for gel III and gel IV are shown in Figure 17. It can be seen that adding Raf fibers to the gel can effectively improve its ability to remain in artificial cracks. In terms of the roughness of the core, the surface roughness is also an important factor affecting the gel’s pressure resistance. Given the limited characterization methods used, this experiment only served to compare the pressure effects of composite hydrogels with different components. In practical leak plugging scenarios, the ideal state for the gel is to withstand formation pressure without gradually creeping under compression. However, if the gel experiences significant creep, it may lead to leak plugging failure or repeated leakage. Based on the two pressure parameters that we defined, there exists a pressure range from the time that the gel starts to creep to the time that the sealing effect is lost. When using PMG in practical applications, the dosage and pressure limits should be fully considered, and it should be used in conjunction with other leak plugging materials to achieve the sealing of the leaking formation.

## 4. Conclusions

The main components of PMG include polyvinyl alcohol, borax, and sodium silicate. Additionally, nylon fibers and dibutyl phthalate are added to enhance the function of the gel. A comparison was made between the performance of PMG and that of commonly used gels in the field. PMG exhibits superior performance in terms of temperature resistance, salt resistance, and erosion resistance. Experiments show that at 60 degrees Celsius, the flowability of PMG increases while maintaining good performance. Under simulated formation water conditions with salinity of 12,500 mg/L, PMG exhibits long-term stability. PMG has minimal dissolution when soaked in water or gasoline for 12 h, and its gel strength remains unchanged.

PMG is a gel designed with the purpose of providing better plugging conditions than other plugging materials by reducing formation leakage rates. The plugging performance of PMG was tested by simulating leakage formations using indoor instruments. In an experiment to delay the fluid leakage rate, mixing PMG with cement slurry at a ratio of 1:1 resulted in a 5.29 times delay in the leakage rate of the cement slurry. When testing PMG’s performance in simulated fractured formations and crack leakage, PMG exhibited good plugging effects. Based on the above tests, PMG can be considered as a material to be used in conjunction with other plugging materials to seal complex water-containing leakage formations such as cracks and karst caves, and its application prospects are very promising.

## Figures and Tables

**Table 1:** Gel Strength Codes.

Code	Gel Type	Description of Gel State
A	No detectable gel formed	The gel appears to have the same viscosity (fluidity) as the original polymer solution and no gel is visually detectable.
B	Highly flowing gel	The gel appears to be only slightly more viscous than the initial polymer solution.
C	Flowing gel	Most of the obviously detectable gel flows to the bottle or ampule top upon inversion.
D	Moderately flowing gel	A small portion (about 5 to 15%) of the gel does not readily flow to the bottle or ampule top upon inversion.
E	Barely flowlng gel	The gel can barely flow to the bottle or ampuletop and or a significant portion (>15%) of the gel does not flow uponinversion.
F	Highly deformable nonflowing gel	The gel does not flow to the bottle or ampule top upon inversion (gel falls slightly short of reaching the bottle or ampule top).
G	Moderately deformable nonflowing gel	The gel flows about halfway down the bottle or ampule upon inversion.
H	Slightly deformable non-flowing gel	Only the gel surface slightly deforms upon inversion.
I	Rigid gel	There is no gel-surface deformation upon inversion and the gel is stable and clear.
J	Ringing rigid gel	A turning-fork-like mechanical vibration can be felt after tapping the bottle—this code is not used during high-temperature ampule testing due to use of the safety shield container.

**Table 2:** Comparison results of alkali treatment.

Composition of PVA Gel	Experimental Operations	Description of Gel Performance
3% PVA + 0.6% borax	/	PH = 8.72, gel has strong fluidity, GSC visual code is C.
	Adjust the pH using sodium hydroxide	With the increase in pH value, the fluidity of the gel decreases obviously when pH = 9.21, until pH = 11.24. The strength grade is D by GSC visual code, and the strength of the gel is 1.12 N.
	Add 1% sodium silicate	PH = 11.27, GSC visual code is H, gel strength is 1.42 N.

**Table 3:** Effect of salinity on PMG.

Salinity/(mg/L)	Volume of Gel/(mL)	Residual Gel/(mL)	Strength after Immersion/(N)	Remarks
0	200	170	6.86	The surface of the gel fragments exposes more fibers, but the gel is still able to heal properly.
5000	200	215	2.55	The gel surface is soft and can heal properly without any dehydration phenomenon.
10,000	200	202	3.15	The gel surface appears whitish but can still heal properly without any dehydration phenomenon.
12,500	200	195	3.6	The gel surface appears whitish but can still heal properly. After 1 day of standing, there is slight dehydration. Slightly dehydrated when left for 1 d.
15,000	200	192	5.17	The gel surface turns white, healing occurs slowly, and, after 1 day of standing, the dehydration rate reaches 70%.
20,000	200	175	5.37	The gel surface turns white, the healing process is slow, and, after 1 day of standing, the dehydration rate reaches 88%.

**Table 4:** Details of core parameters.

Core Number	Entrance Width/ (mm)	Inlet Length/ (mm)	Outlet Width/ (mm)	Outlet Length/ (mm)	Remarks
I	3.8	19.0	1.0	16.0	Smooth surface
II	2.8	21.0	2.0	20.0	Smooth surface
III	4.0	23.0	1.2	20.0	Rough surface

## Data Availability

The authors are not prepared to disclose data due to privacy reasons.

## References

[1] Alkinani H.H., Al-Hameedi A.T.T., Dunn-Norman S. State-of-the art review of lost circulation materials and treatments-part II: Prob- ability and cost analyses. Proceedings of the International Petroleum Technology Conference.

[2] Wang W., Liu Q., Guo X., Wang X. (2019). Review of lost circulation prevention and plugging technilogy in Tahe Oilfield. Explor. Eng. (Rock Soil Drill. Tunneling).

[3] Su X., Lian Z., Fang J., Xiong H., Wu R., Yuan Y. (2019). Lost circulation material for abnormally high temperature and pressure fractured-vuggy carbonate reservoirs in Tazhong block, Tarim Basin, NW China. Pet. Explor. Dev..

[4] Yang C., Wu S., Ping S., Liu C., Jing Y. (2021). Indoor development and evaluation of high-temperature resistant pressure-bearing composite plugging gel. Liaoning Chem. Ind..

[5] Ji Y., Han L., He B., Gao L., Pu Y. (2015). Application of gel plugging agent ZND-2 for complex well leak treatment in Amu Darya test gas. Drill. Prod. Technol..

[6] Magzoub M.I., Salehi S., Hussein I.A., Nasser M.S. (2019). Loss circulation in drilling and well construction: The significance of applications of crosslinked polymers in wellbore strengthening: A review. J. Pet. Sci. Eng..

[7] Xiao J. (2018). Halliburton’s SentinelCem^TM plugging cement slurry. Pet. Drill. Technol..

[8] Chen Z., Wang Y., Li D., Liu S., Lin Y. (2015). Application of high temperature resistant downhole cross-linked cement slurrying plugging technology in Tahe oilfield. Drill. Complet. Fluids.

[9] Li W., Yutong Y.B., Yutong L.I. (2021). Research and application progress of drilling fluid lost circulation materials and technical countermeasures for lost circulation control. Sci. Technol. Eng..

[10] Wu X. (2023). Research on high-temperature resistant multi-layered expanded graphite materials and their applications. Drill. Eng..

[11] Chen K., Zhang X., Liu X., Wang J., Li Z. (2019). Preparation and performance of self-adhesive particles based on fissure-type reservoirs. Synth. Resins Plast..

[12] Adewunmi A.A., Ismail S., Sultan A.S. (2017). Investigation into the viscoelastic response at various gelation performance, thermal stability and swelling kinetics of fly ash reinforced polymer gels for water control in mature oilfields. Asia-Pac. J. Chem. Eng..

[13] Zhou J. (2017). Study on the Technique of Solidifiable Plugging Fluid for the Fractured Lost Circulation Formation. Ph.D. Thesis.

[14] Alsaba M., Dushaishi M.f.A., Nygaard R., Nes O.M., Saasen A. (2016). Updated Criterion to Select Particle Size Distribution of Lost Circulation Materials for an Effective Fracture Sealing. J. Pet. Sci. Eng..

[15] Abrams A. (1977). Mud Design to Minimize Rock Impairment Due to Particle Invasion. J. Pet. Technol..

[16] Liu W. (2019). Reservoir Protection Technology for Fractured Tight Sandstone of Sulige Gasfield. Ph.D. Thesis.

[17] Li D.Q. (2012). Numerical and Experimental Investigations of Drilling Fluid Losses in Fractured Formations. Ph.D. Thesis.

[18] Sun J., Bai Y., Cheng R., Lyu K., Liu F., Feng J., Lei S., Zhang J., Hao H. (2021). Research progress and prospect of plugging technologies for fractured formation with severe lost circulation. Pet. Explor. Dev..

[19] Liu J., Liu S., Long D., Chen C., Jin R. (2017). Strengthening Plugging Operations by Combining Cross-Linked Film and Chemical Consolidation in Well Ming-1. Pet. Drill. Technol..

[20] Han C., Luo M., Yang Y., Liu X., Li W. (2019). Key drilling technologies for HTHP wells with narrow safety density window in the Yingqiong Basin. Pet. Drill. Prod. Technol..

[21] Lin H.L., Liu Y.F., Yu T.L., Liu W.H., Rwei S.P. (2005). Light scattering and viscoelasticity study of poly (vinyl alcohol)–borax aqueous solutions and gels. Polymer.

[22] Mahjoub H.F., Zammali M., Abbes C., Othman T. (2019). Microrheological study of PVA/borax physical gels: Effect of chain length and elastic reinforcement by sodium hydroxide addition. J. Mol. Liq..

[23] Huang P.-L. (2016). Application of Polyvinyl Alcohol Modified by Sodium Silicate. Ph.D. Thesis.

[24] Sydansk R.D. (1993). Acrylamide-polymer/chromium(III)-carboxyl-ategels for near wellbore matrix treatments. SPE Adv. Technol. Ser..

[25] Li J. (2021). Chain Conformation and Dynamics of Poly(vinyl alcohol) and Borax Complexing System. Ph.D. Thesis.

